# Introduction to the special issue: Tree invasions: towards a better understanding of their complex evolutionary dynamics

**DOI:** 10.1093/aobpla/plx014

**Published:** 2017-04-05

**Authors:** Heidi Hirsch, David M. Richardson, Johannes J. Le Roux

**Affiliations:** Centre for Invasion Biology, Department of Botany & Zoology, Stellenbosch University, Matieland 7602, South Africa

**Keywords:** Biological invasions, evolutionary mechanisms, rapid evolution, tree invasions

## Abstract

Many invasive plants show evidence of trait-based evolutionary change, but these remain largely unexplored for invasive trees. The increasing number of invasive trees and their tremendous impacts worldwide, however, illustrates the urgent need to bridge this knowledge gap to apply efficient management. Consequently, an interdisciplinary workshop, held in 2015 at Stellenbosch University in Stellenbosch, South Africa, brought together international researchers to discuss our understanding of evolutionary dynamics in invasive trees. The main outcome of this workshop is this Special Issue of *AoB PLANTS*. The collection of papers in this issue has helped to identify and assess the evolutionary mechanisms that are likely to influence tree invasions. It also facilitated expansion of the unified framework for biological invasions to incorporate key evolutionary processes. The papers cover a wide range of evolutionary mechanisms in tree genomes (adaptation), epigenomes (phenotypic plasticity) and their second genomes (mutualists), and show how such mechanisms can impact tree invasion processes and management. The special issue provides a comprehensive overview of the factors that promote and mitigate the invasive success of tree species in many parts of the world. It also shows that incorporating evolutionary concepts is crucial for understanding the complex drivers of tree invasions and has much potential to improve management. The contributions of the special issue also highlight many priorities for further work in the face of ever-increasing tree invasions; the complexity of this research needs calls for expanded interdisciplinary research collaborations.

## Teaser

This short communication provides an overview of the articles contained in the AoB PLANTS Special Issue, Evolutionary dynamics of tree invasions. The articles cover a wide range of evolutionary mechanisms in tree genomes, epigenomes, and their second genomes, and enhance our understanding and the conceptualization of the complex evolutionary drivers of tree invasions. Additionally, this AoB PLANTS Special Issue identifies directions for further research in the face of the increasing impact of invasive trees worldwide, and calls for interdisciplinary research collaborations.

## Introduction

Plants introduced by humans to areas well outside their native ranges must confront multiple selective barriers that influence their capacity to become invasive (Richardson *et al.* 2000*b*, 2011; Blackburn *et al.* 2011). Many invasive plant species show evolutionary responses to these selective pressures along the introduction–naturalization–invasion (INI) continuum (Richardson and Pyšek 2012), and this can result in phenotypic, physiological or ecological divergence from source populations in the native range (Prentis *et al.* 2008; Whitney and Gabler 2008; Turner *et al.* 2014). The invasion success of a species can further be influenced by events in its pre-introduction evolutionary history which have shaped its plasticity, adaptability, or which may have led to pre-adaptations (Lee 2010). For example, Guo *et al.* (2014) illustrated that a combination of pre- and post-introduction evolution has contributed to the invasion success of *Phragmites australis* in North America (Guo *et al.* 2014). Specifically, Guo *et al.* (2014) found that pre-adapted ecophysiological traits mediated the invasion of introduced *P. australis* and that post-introduction evolution in photosynthesis- and growth-related traits further benefitted its invasiveness. Most of our knowledge about the role and dynamics of evolutionary processes during plant invasions, however, comes from studies on relatively short-lived species like herbaceous annuals, while information on processes affecting trees is much scarcer. This is attributed to the long lifespans and generation times of trees which makes inferences about evolutionary dynamics over multiple generations difficult (Zenni *et al.* 2017). However, the rapidly increasing number of invasive tree species and the escalation in the overall extent and types of their impacts worldwide highlights their importance as damaging invaders (Richardson and Rejmánek 2011; Rejmánek and Richardson 2013). There is increasing evidence that invasive tree populations can exhibit morphological or physiological traits, or ecological interactions (e.g. symbiotic relationships), that differ substantially from those in the native range (Gundale *et al.* 2014; Heberling *et al.* 2016). A long history of forestry studies (e.g. provenance trials) has helped us to gain an initial understanding of the potential evolutionary mechanisms (e.g. standing genetic variation, hybridization, adaptation) which can contribute to these changes (e.g. Yazdani *et al.* 1985; Stettler and Bradshaw 1994; Hamrick 2004; Zenni *et al.* 2017). However, forestry studies focus mainly on tree improvement and not on the role of evolutionary mechanisms that occur during progression along the INI continuum when trees escape cultivation and potentially become invasive. Consequently, it is crucial to expand our knowledge of evolution in invasive trees, not only to enhance our understanding of invasion ecology, but also to guide management practices. Evolutionary studies on tree invasions are also interesting to ecologists and evolutionary biologists because they are unique natural experiments that afford outstanding opportunities for understanding evolution during invasions as well as during general colonization processes (e.g. see Richardson *et al.* 2004; Gundale *et al.* 2014; Bock *et al.* 2015; Zenni *et al.* 2016). Invasive trees have several key characteristics that distinguish them from other invasive plants and which can have implications for their evolutionary dynamics:

- Their unique architecture, long life cycles and high reproductive output can have a strong influence on the mode and rate of evolutionary processes (Petit and Hampe 2006).
- Introduction history. Most invasive tree species were intentionally introduced for forestry, food production and agroforestry purposes (Richardson and Rejmánek 2011). Because of their varied uses, many species are introduced in very large numbers and over large areas, ensuring high propagule pressure and thus genetic diversity; this often helps in overcoming selective barriers and results in the fast-tracking of local adaptation (Wilson *et al.* 2009; Donaldson *et al.* 2014).
- Artificial selection. Intentionally introduced tree species are often subject to selection for desirable traits like vigour, growth rates or high tolerance of certain environmental conditions such as disease; these traits are often associated with invasiveness (Le Roux *et al.* 2011; Donaldson *et al.* 2014).
- Symbiotic interactions. Most tree species rely on symbiotic interactions of which those involving roots and rhizosphere soil microorganisms (e.g. nitrogen-fixing rhizobia, mycorrhizal fungi) are especially important (Richardson *et al.* 2000*a*). Such closely associated microbial mutualists, also referred to as ‘second genomes’ (Zenni *et al*. 2017), can be crucial for plant growth and health, and potentially act as drivers of evolution (Berg *et al.* 2014; Rosenberg and Zilber-Rosenberg 2016).
- Introduced trees are often planted in monotypic stands covering large geographical areas in their non-native ranges. On the one hand, this exposes them to spatially heterogenic environmental conditions which can promote local adaptation (Richardson *et al.* 2004; Zenni *et al.* 2014). On the other hand, such stands may attract antagonists (i.e. pests and diseases) which may induce natural selection (Jactel *et al.* 2005).

Despite the unique circumstances and characteristics outlined above that often underlie tree invasions, very scant information is available in the primary literature on their evolutionary dynamics compared with other taxa, and generality is certainly lacking. A workshop held at Stellenbosch University in Stellenbosch, South Africa, in November 2015 brought together 25 researchers from many parts of the world representing disciplines that are rarely integrated: genomic evolution, plant–mutualist interactions, pathology and invasion ecology. The aims of the 2-day meeting were to synthesize and extend our current knowledge on the evolutionary mechanisms in tree genomes, epigenomes and their second genomes. The workshop resulted in a joint publication (Zenni *et al.* 2017) that emerged during the discussion sessions and 12 additional articles which cover a wide range of evolutionary mechanisms in invasions of introduced trees. Early versions of these articles were presented and discussed during the workshop and now, after peer review and revision, form this special issue of *AoB PLANTS*.

## Key Insights from the Special Issue

**Figure 1:**
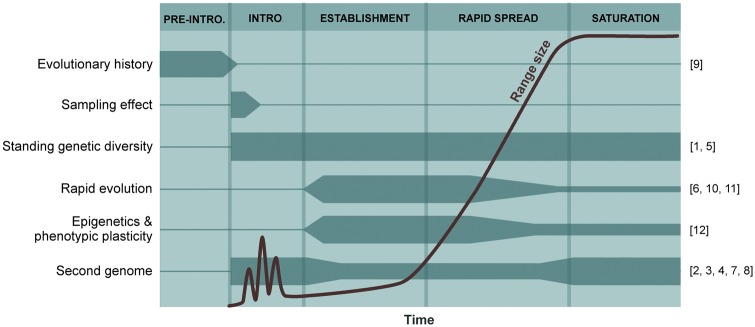
Evolutionary mechanisms involved in tree invasions and their changing importance during key invasion stages (the thickness of horizontal bars indicates relative importance). The key stages are defined according to the non-native range size change over time (curve) after introduction (Intro.) as shown by Prentis *et al.* (2008). The depicted evolutionary mechanisms were compiled by Zenni *et al.* (2017). For the sake of convenience, the category ‘standing genetic diversity’ represents ‘founder effects’ and ‘admixture, hybridization and polyploidization’ which are separately discussed by Zenni *et al.* (2017). For the same reason, the category ‘genotype-by-environment interactions’ defined by Zenni *et al.* (2017) is, here, considered under ‘rapid evolution’. Numbers in square brackets on the right side of the figure denote the papers in the special issue that deal with the corresponding evolutionary mechanisms: [1] Besnard and Cuneo (2016); [2] Burgess *et al.* (2016); [3] Crous *et al.* (2017); [4] Dickie *et al.* (2016); [5] Gaskin (2017); [6] Hirsch *et al.* (2016); [7] Klock *et al.* (2016); [8] Le Roux *et al.* (2016); [9] Miller *et al.* (2017); [10] Siemann *et al.* (2017); [11] Zenni *et al.* (2016); [12] Zimmermann *et al.* (2016).

Despite their significant impact on overcoming barriers along the INI continuum, evolutionary mechanisms were missing from frameworks used in conceptualizing aspects of biological invasions. Zenni *et al.* (2017) sought to populate the unified framework for biological invasions proposed by Blackburn *et al.* (2011) with the potential evolutionary mechanisms that can affect each of the invasion stages and barriers for introduced trees. The aim of this review was to evaluate how these mechanisms impact, positively and/or negatively, tree invasions along the INI continuum. The following evolutionary mechanisms underlying tree invasions were identified and integrated in the unified framework: pre-introduction evolutionary history; sampling effect; the influence of founder effects, admixture, hybridization and polyploidization on genetic diversity (hereafter termed ‘standing genetic diversity’); genotype-by-environment interactions; rapid evolution; epigenetics (phenotypic plasticity); and second genomes. Here, we firstly summarized these evolutionary mechanisms and their relative importance during different stages along the INI continuum (Fig. 1), and secondly, introduce the 12 papers of the special issue by assigning them to the applicable evolutionary mechanism identified and described in detail by Zenni *et al.* (2017) (Fig. 1).

### Pre-introduction evolutionary history

Evolutionary history within its native range can determine whether a species will become invasive when introduced to a novel area (Marsico *et al.* 2010). Miller *et al.* (2017) investigated such pre-introduction factors in tree invasions by determining whether differences in the introduction status (introduced, naturalized, invasive) can be explained by relatedness (phylogeny) and biogeography (native range size parameters). The authors considered two genera of Australian trees, *Acacia* and *Eucalyptus* (*sensu lato*)—groups which are interesting to invasion ecologists for different reasons. For example, almost all introduced acacia species that have been widely planted have become highly invasive whereas only a few eucalypt species are highly invasive. The authors found that in both genera the introduction status (introduced, naturalized and invasive) of species showed no phylogenetic signal, except for introduced acacias. Therefore, the placement of taxa along the INI continuum appears to be phylogenetically random. On the other hand, range size appears to increase in both *Acacia* and *Eucalyptus* from introduced to naturalized to invasive. Although the native range size for species of both genera increases with invasiveness status (species with larger ranges are more likely to be invasive), the level of aggregation (in their native ranges), as measured by the scale-occupancy curves, was different, decreasing for *Acacia* (higher rate of spread), but increasing for *Eucalyptus* (lower rate of spread) along the INI continuum.

### Standing genetic diversity

Standing genetic diversity must be important during the progression of non-native species along the INI continuum as strong selection is expected under novel environmental conditions (Bock *et al.* 2015) (Fig. 1). Several processes can impact genetic diversity, either by reducing it (i.e. founder effects) or by counteracting reductions associated with introduction (i.e. admixture, hybridization and polyploidization) (Bock *et al.* 2015). Two special issue contributions focused on such evolutionary mechanisms that can rescue invasive tree species from the potential negative effects of reduced genetic diversity.

Besnard and Cuneo (2016) reviewed the history and ecology of invasive olives (*Olea* spp.). Two subspecies, European olive (*O. europaea* ssp. *europaea*) and African olive (*O. europaea* ssp. *cuspidate*), are a superb study system for tree invasions due to their parallel invasions in different climatic zones of Australia. The authors point out that the two olive subspecies had different introduction histories: multiple introductions for European olive which helped to maintain high genetic diversity vs. successive bottlenecks in the African olive which resulted in reduced genetic diversity. Moreover, genetic admixture between the subspecies occurred early after their introduction into Australia. The review provides comprehensive directions for future research on these invasive olives and shows how such research can increase our understanding of the genetic basis of tree invasions.

In his review, Gaskin (2017) assessed the prominence and role of hybridization during invasion processes of trees. He found evidence of 20 hybrid invasive tree taxa. Importantly, in seven of these taxa researchers identified phenotypes that make the hybrids better invaders than their parental species. Also, all hybrid taxa involved intentional introductions of either one or more parental species, or the hybrid itself. This is the first review of hybridization and its link with invasiveness to focus on trees. It also highlights that hybridization events can hamper management efforts because invasive hybrids may lack co-evolved biological control agents. Such a lack of control opportunities may lead to an unrestricted spread of hybrid phenotypes which may be more vigorous, and therefore of increased impact, than their parental species.

### Rapid evolution

An extensive body of literature shows that non-native plant species can undergo rapid evolutionary changes in response to novel selection pressures in their new ranges (Whitney and Gabler 2008; Buswell *et al.* 2010; Lee 2010). Numerous ecological hypotheses have been postulated to underlie such contemporary evolution. For example, the evolution of increased competitive ability (EICA) hypothesis postulates that, due to the release of co-evolved and specialist natural enemies during introduction into the new range (enemy release hypothesis; Keane and Crawley 2002), introduced plants may reallocate resources associated with enemy defence towards higher investment in growth and reproduction traits (Blossey and Notzold 1995; Felker-Quinn 2013). Rapid evolutionary responses can consequently be assumed to be most important for a non-native plant during its establishment and spread phases, when new selective pressures are most likely to be encountered (Fig. 1). Three contributions to the special issue addressed aspects of rapid evolution during the range expansion of invasive trees by applying common environment or *in situ* experiments.

Hirsch *et al.* (2016) investigated shifts in seedling growth performance between native and non-native populations of the invasive elm, *Ulmus pumila*, under different water and temperature conditions. Traits such as seedling growth, associated with the early parts of a plant's life-cycle, can play an important role during the colonization of new sites. *Ulmuspumila* was introduced to several regions outside its native Asian range and has become naturalized or invasive in most of these regions. A greenhouse experiment simulated different temperature and water stress treatments. Under all treatments, non-native populations from Argentina and the western United States produced more biomass and had enhanced resource allocation to aboveground biomass compared with native populations from Asia. The authors conclude that the enhanced growth performance may contribute to the invasion success of *U. pumila*, at least in areas with low competition from native species. The study shows that experimental approaches focusing on life-history traits important in the early stages of colonization (i.e. germination and seedling growth) can enhance our understanding of tree invasion processes.

A study by Zenni *et al.* (2016) demonstrated the role of rapid evolution in the invasion success of the non-native loblolly pine, *Pinus taeda*, in Brazil, where the species has escaped from plantations. The authors found evidence of rapid evolution in growth rates and a lack of trade-offs between growth and defence traits. It can be assumed that this contemporary evolution facilitated the ability of *P. taeda* populations to cope with the new environmental conditions.

Siemann *et al.* (2017) tested the EICA hypothesis using *Triadica sebifera*, a tree which is an aggressive invader of different ecosystems in the southeastern United States. The authors grew *T. sebifera* plants from native and introduced populations with insect suppression treatments in common gardens in three geographic venues that varied in *T. sebifera* status (native, casual alien, invasive) and insect herbivore communities. The results showed that herbivore damage was highest in the native range. Further, more rapid aboveground growth rates contributed to *T. sebifera*’s success in both the invasive and native ranges independent of aboveground herbivory. Together with strong variation among sites this indicates that plants from invasive populations may only have a strong advantage in a subset of sites in their invasive ranges.

### Second genome

The loss, co-introduction or subsequent accumulation of closely associated microbial communities can promote or confine the invasion success of non-native plants (Richardson *et al.* 2000*a*; Pringle *et al.* 2009; Gundale *et al.* 2014). Dynamics in such symbiotic relations can therefore be considered as most important during the introduction stage (i.e. loss or co-introduction of symbiotic partners) but also at later stages (i.e. accumulation of new symbiotic partners) along the INI continuum (Fig. 1). Five special issue contributions helped to elucidate how such second-genome dynamics can impact tree invasions. The first three contributions focus on the role of mutualist associations during the invasion process and the last two contributions highlight the role of co-introduced or newly accumulated harmful associations (i.e. pathogens and herbivores).

Dickie *et al.* (2016) used two extensive databases of plant-fungal sporocarp associations to understand how the symbiotic ectomycorrhizal associations of native and introduced trees in New Zealand and the United Kingdom differed. These data indicated a restructuring of interactions towards less modular networks in both countries and that functional diversity in symbioses is lower in introduced compared with native trees in New Zealand. This extensive assessment of symbiotic interaction network function and structure at national scales shows how physiological function of trees may be modified, not only by genomic shifts, but also by shifts in the entire assembly of their associated second genomes.

Klock *et al.* (2016) assessed whether the invasion of Australian *Acacia* species in California was influenced by their promiscuity with rhizobial symbionts. The authors paired *Acacia* species with different introduction statuses (introduced, naturalized, invasive) in California with soils containing different rhizobial communities, and examined whether invasive acacias could form more effective symbioses with a greater diversity of rhizobial strains than introduced and naturalized acacias. Contrary to previous research (Klock *et al*. 2015), the results showed no differences in host-promiscuity among invasiveness categories. A reason for this may be that all *Acacia* species that were examined are invasive in at least one part of the world (though they differ in invasiveness in California) and are therefore promiscuous hosts.

In their study, also on Australian acacias, Le Roux *et al.* (2016) examined the structure of native legume-rhizobium interaction networks and how these change in response to invasions. For this the authors collected acacias, native legumes, and their associated rhizobia along a gradient of invasion in South Africa’s Cape Floristic Region. Rhizobia were isolated from all legumes and their identities determined using DNA barcoding. Constructed interaction networks showed that invasive acacias do not infiltrate existing native legume-rhizobium networks but that they form associations with a unique subset of rhizobia that are not associated with native legumes, i.e. strong modularity. These results are in stark contrast to other types of mutualistic interaction networks (e.g. seed dispersal and pollination) indicating that invasive plants usually infiltrate networks through generalized interactions. Le Roux *et al.* (2016) concluded that their findings may reflect co-invasion of legumes and their rhizobia.

Burgess *et al.* (2016) conducted a mini-review to explore the different fungal associates which arrive with non-native trees. The results show that invasive success of trees can vary depending on the different categories of fungal associates. Beneficial symbiotic fungi can assist the establishment of their host trees, but may not form novel associations with native trees in the new range. In contrast, parasitic fungi could potentially reduce the invasion success of their hosts, with the potential to move onto new native hosts (hosts shifts). Although the frequency of identified host shifts was low, and depended upon the fungal guild, when such shifts occur they can be devastating for native hosts.

To investigate the dimensions of the ecological disequilibrium caused by the separation of non-native trees and their natural enemies (pathogens and insects), Crous *et al.* (2017) conducted a review on long-established, non-native tree plantations in South Africa. They assessed the accumulation of non-native pathogens and insect pests onto planted *Acacia*, *Eucalyptus* and *Pinus* species, but also examined native (South African) pathogens and insects that utilized species in these genera. Importantly, the phylogenetic relatedness between non-native and native floras appears to influence the likelihood of pathogen shifts among them. This was not the case for insects. Crous *et al.* (2017) concluded that when multiple tree species are introduced one should expect considerable spatial and temporal variation in ecological disequilibrium conditions among taxa, a result that has implications for biosecurity and other management practices.

### Epigenetics and phenotypic plasticity

Epigenetics involves phenotypic variation due to variation in gene expression that is not linked to variation in gene sequences, but to other molecular mechanisms such as DNA and RNA methylation and histone modification (Willyard 2017). Epigenetic variation is thus tightly associated with phenotypic plasticity and may be heritable over multiple generations (Duncan *et al.* 2014). For non-native plants, such plastic responses can be crucial for overcoming novel environmental conditions that are experienced, especially when genetic diversity is low (Rollins *et al.* 2013). Along the INI continuum, epigenetic mechanisms (i.e. phenotypic plasticity) are assumed to be most important during establishment and range expansion when rapid responses towards the new environmental conditions are essential (Fig. 1). However, our knowledge of the role of epigenetics mechanisms during plant invasion processes is still very poor; in the case of tree invasions almost nothing is known. Invasive tree species in which invasion success is largely facilitated by phenotypic plasticity could provide ideal study systems for bridging this knowledge gap.

Zimmermann *et al.* (2016) investigated phenotypic responses in traits associated with the invasion of *Casuarina equisetifolia* in sites with stressful environmental conditions (i.e. high temperature, solar radiation, drought and salinity) in Brazilian sandy coastal plains. They postulated that a low level of phenotypic plasticity would be beneficial in habitats with multiple stress factors. Considering that the phenotype is the result of the integration of characters in each environmental condition, phenotypic integration (the pattern and magnitude of correlation among different plant traits) may constrain phenotypic plasticity. *Casuarina equisetifolia* exhibited high germination plasticity, although plasticity in traits of seedlings was low. The authors argue that the positive effect of phenotypic integration on the plastic expression of morphological traits in shade is a key factor that allows *C. equisetifolia* to invade in the Brazilian sandy coastal plains.

## Concluding Remarks and Future Directions

The contributions in this Special Issue have helped us to merge contemporary questions, experimental data, and ideas from multiple perspectives to shed new light on the evolutionary dynamics of invasive trees. Moreover, the Special Issue highlights the multi-trophic aspects of tree invasions, in that trees might not arrive alone (e.g. endophytes), but even worse, in time would attract an extensive array of non-native organisms that may or may not become invasive. We have learned that incorporating evolutionary concepts is crucial for understanding the complex drivers of tree invasions and that such information can improve management.

The papers summarized here have also identified priorities for further work in the face of ever-increasing tree invasions. For example, there is much scope to utilize long-term forestry plantations which provide superb study systems for improving our understanding of the evolutionary mechanisms that enable many tree species trees to escape cultivation and spread. Shorter-term experiments, comparing native and non-native tree populations under common environmental conditions, can also shed light on potential evolutionary shifts in early life-cycle traits that are important for the successful colonization of new areas during the spread of an invasive tree species. Several contributions to the Special Issue show that second genomes are a crucial mediator of invasive success in trees. Relatively few studies have been conducted in this area, and such research definitely needs to be intensified on several model systems to obtain more empirical data on the role of second genomes during tree invasions. We know of no studies on epigenetic mechanisms during tree invasions (but see Bräutigam *et al.* 2013; Guarino *et al.* 2015), and such insights would greatly enhance our understanding of phenotypic plasticity and why many non-native tree species are such successful invaders despite having low genetic diversity (the ‘genetic paradox’). The complexity and diversity of the research needed to unravel such issues calls for enhanced transversal collaborations involving researchers from disciplines like invasion ecology, microbiology, forestry, plant pathology, mycology and plant genetics. 

## Sources of Funding

The workshop on ‘Evolutionary dynamics of tree invasions’ was hosted by the DST-NRF Centre of Excellence for Invasion Biology (C•I•B) in Stellenbosch, South Africa, in November 2015. Funding was provided by the C•I•B, Stellenbosch University (through the office of the Vice Rector: Research, Innovation and Postgraduate Studies), and the South African National Research Foundation (grants 98182 to JLR and 85417 to DMR).

## Contribution by the Authors

H.H. led the writing but all authors contributed equal editorial advice to this article.

## Conflict of Interest Statement

None declared.
